# Investigating the inhibitory effect of miR-34a, miR-449a, miR-1827, and miR-106b on target genes including NOTCH1, c-Myc, and CCND1 in human T cell acute lymphoblastic leukemia clinical samples and cell line

**DOI:** 10.22038/IJBMS.2019.40695.9615

**Published:** 2020-03

**Authors:** Tohid Naderi, Samira Mohammadi-Yeganeh, Neda Mohammadi-Hezaveh, Razie Hadavi, Ahmad Gharehbaghian, Nader Vazifeh-Shiran, Vahid Fallah Azad, Mahdi Paryan

**Affiliations:** 1 Department of Laboratory Hematology and Blood Bank, School of Allied Medicine, Shahid Beheshti University of medical sciences, Tehran, Iran; 2 Medical Nanotechnology Research Center, Shahid Beheshti University of Medical Sciences, Tehran, Iran; 3 Department of Biotechnology, School of Advanced Technologies in Medicine, Shahid Beheshti University of Medical Sciences, Tehran, Iran; 4 Department of Biochemistry, School of Medicine, Semnan University of Medical Sciences, Semnan, Iran; 5 Cellular and Molecular Biology Research Center, Shahid Beheshti University of Medical Sciences, Tehran, Iran; 6 Mahak Charity Hospital, Tehran, Iran; 7 Department of Research and Development, Production and Research Complex, Pasteur Institute of Iran, Tehran, Iran

**Keywords:** Bioinformatics, Biomarker, miRNA, Notch signaling pathway T-cell acute lymphoblastic, leukemia

## Abstract

**Objective(s):**

microRNAs are small non-coding molecules that regulate gene expression in various biological processes. T-cell acute lymphoblastic leukemia (T-ALL) is a malignancy accompanied with genetic aberrations and accounts for 20% of children’s and adult’s ALL. Notch signaling pathway dysregulation occurs in 60% of T-ALL cases. In the present study, we aimed to determine the relationship between miRNAs and genes involved in Notch signaling pathway.

**Materials and Methods:**

Considering the role of the pathway and its down-stream genes in proliferation, differentiation, cell cycle, and apoptosis, *NOTCH1, c-Myc*, and *CCND1* genes were selected as target genes. Using bioinformatics studies, miR-34a, miR-449a, miR-1827, and miR-106b were selected as miRNAs targeting the above-mentioned genes. We evaluated these genes and miRNAs in T-ALL clinical samples as well as Jurkat cell line, in which NOTCH1 is overexpressed.

**Results:**

Quantitative Real-Time PCR indicated that *NOTCH1, c-Myc, *and *CCND1* were overexpressed in samples with decreased expression of miR-34a. In addition, we observed that samples with decreased expression of miR-449a showed increased expression of *NOTCH1* and *CCND1*. Furthermore, we analyzed the expression of miR-1827 and miR-106b, which target *c-Myc* and *CCND1*, respectively. We found out that the expression of miR-1827, miR-106b, and their respective target genes were inversely correlated in 80% and 75% of the cases (r=0.8), respectively. Furthermore, in Jurkat cell line, the expression of target genes was increased while the candidate miRNAs except miR-34a were decreased.

**Conclusion:**

These miRNAs can be proposed as biomarkers and new therapeutic targets in T-ALL patients who have *NOTCH1* overexpression.

## Introduction

T-cell acute lymphoblastic leukemia (T-ALL) is an invasive and malignant cancer of immature thymocytes that accounts for 10-15% of T-ALL in children and 25% of adult’s T-ALL. The prevalence of the disease in men is three times more than that of women. Affected patients have elevated white blood cells count, neurological complications, and mediastinal mass. Compared to B-cell complications, T-ALL occurs in older ages and is more invasive (1). It is induced by multiple oncogenic stimuli and genetic aberrations, which lead to increased cell proliferation and survival as well as inhibition of T-cell progenitor’s differentiation (2). One of the main dysregulated signaling pathways implicated in T-ALL is Notch, which is related to the development and drug resistance of the disease (3). This pathway regulates scores of cellular processes such as proliferation, differentiation, apoptosis, replication, cell survival, and developmental processes like neurogenesis and vasculogenesis (4). In addition, Notch signaling pathway plays a pivotal role in self-renewal of hematopetic stem cells and hematopoiesis, leading to generation of all types of blood cells (5). The principal role of Notch is determination of T-cells fate, progression of differentiation, T-Cell Receptor (TCR) signaling, and T-cell immune functions in that it is necessary for the differentiation of double negative T-cells (DN1) to early thymocyte progenitors (ETPs) and also for the differentiation of double positive T-cells (DP) (6). Aberrant activation of Notch signaling resulting from various mutations has been observed in neuroblastoma (7), prostate cancer (8), breast cancer (9), glioblastoma (10), and leukemia (11). In addition, mutations of *NOTCH1* have been discovered in nearly 60% of T-ALL patients, which underpins the importance of aberrant activation of *NOTCH1* in leukemogenesis (12).

*MYC* and *CCND1* are two genes in Notch signaling that encode c-Myc and Cyclin-D1, respectively. C-Myc is a transcription factor inhibitor, and by suppressing Cip, Kip, INK4 proteins, and their inhibitory function, results in increased proliferation (13). Cyclin-D1 is the regulator of cyclin-dependent kinases (CDKs) in cell cycle that has a prominent role in the initiation of G1 phase. Therefore, any anomalies in Cyclin-D1 increase the chance of cancer development. Several studies have demonstrated its role in small cell lung cancer (14), bladder cancer (15), pancreas cancer (16), breast cancer, etc. (17).

One of the regulatory molecules of these proteins are microRNAs (miRNAs), which are small non-coding molecules (19-24 nucleotides length) and key regulators of differentiation, proliferation, and survival of the cells (18). These molecules regulate the expression of genes by complementary or semi-complementary binding to their target mRNAs. Based on the region of complementarity in mRNAs, i.e. 3’-untralslated region (3’-UTR), 5’-UTR, or coding sequence (CDS), they can increase or decrease gene expression during translation. However, 3’-UTR is the most usual target region, and miRNA binding to it results in translation inhibition (18, 19). Nonetheless, some miRNAs are oncogenic, and some are tumor suppressors. The function of miRNAs can be affected by deletions, point mutations, epigenetic silencing, and splicing (20).

In addition to specific genetic modifications, DNA methylation, and gene expression patterns, miRNA expression pattern can also elicit informative clinical data for physicians. Since being stable in clinical samples and body fluids such as serum, saliva, and urine, miRNAs can be used as more useful prognostic biomarkers than mRNAs. Their role in various cancers has been proved, and they can be used as prognostic markers and therapeutic targets as well (21).

Today, several methods like microarray, deep sequencing, and bioinformatics algorithms are used to determine the expression profile of miRNAs in various diseases. The aim of the current research was to predict miRNAs targeting NOTCH1, c-Myc, and CCND1 mRNAs using bioinformatics methods and to determine their expression in Jurkat cell line and T-ALL clinical samples. 

## Materials and Methods

***Clinical samples***

Since overexpression of Notch signaling pathway is one of the causes of T-ALL, patients with upregulation of Notch gene that is an indicative of Notch signaling pathway dysregulation were selected in this study. Twenty bone-marrow clinical samples, previously diagnosed as T-ALL were received from Payvand Laboratory (Tehran, Iran) in which 12 samples had been diagnosed with increased expression of *NOTCH1*. Seventeen clinical samples were also obtained from Transplantation Laboratory (Cell bank of Mahak hospitals, Tehran, Iran). These samples were analyzed for *NOTCH1* expression level, and finally 8 of 17 were approved to be *NOTCH1* overexpressed. Totally, 20 clinical samples with increased expression of *NOTCH1* were used for this study. In addition, 15 peripheral blood samples from healthy volunteers who had normal blood- and cancer-related indices were used as normal controls. The written informed consents were received from all patients and healthy volunteers. This study was under the supervision of ethics committee of Shahid Beheshti University of Medical Sciences (Ethics code: IR.SBMU.RETECH.REC.1396.1311)

***Cell culture***

Jurkat cell line (T-ALL cell line, CRL-1900) was used since it shows an increased expression of *NOTCH1*. The cell line was obtained from cell bank of Stem Cell Technology Research Center (Tehran, Iran). Frozen Jurkat cell line were thawed and cultured in RPMI 1640 containing 100 mg/ml penicillin/streptomycin (Dana pharmaceuticals, Tabriz, Iran) and 10% fetal bovine serum (FBS) and were incubated in an humidified atmosphere at 37 ^°^C with 5 percent CO_2_.

***Gene selection and Bioinformatics prediction of targeting miRNAs***

After thorough review of T-ALL and Notch signaling pathway and related research articles, *NOTCH1*, *c-Myc*, and *CCND1* were selected as key genes in T-ALL.

miRNAs targeting these genes were predicted using different programs and databases such as TargetScan, PicTar, MiRanda, DIANA microT CDS, miRBase, and mirWalk. Researchers can find a list of targeting miRNAs for a given gene by applying these databanks and software. miRNAs are predicted based on criteria such as species, tissue, target gene sequence, strength and type of binding to seed region, nature of miRNA: mRNA binding, etc. TargetScan as the most powerful prediction program was used for final confirmation of the selected miRNAs. After analyzing the results, miR-34a and miR-449a were selected that target all three target genes. Furthermore, miR-1827 and miR-106b that target c-Myc and CCND1 mRNAs respectively were selected due to high miRNA: mRNA attachment scores.

***Primer design for the genes and miRNAs***

The sequences of the genes were retrieved from NCBI (https://www.ncbi.nlm.nih.gov/gene/). AlleleID7 software was used for designing primers for exon junctions. In case of existence of mRNA variants, all transcripts were aligned using MEGA6 software, and then primers were designed for conserved sequences. Finally, the primers were analyzed using NCBI BLAST. 

Since miRNAs are short sequences, their amplification with regular primers is not practical. To overcome this obstacle, stem-loop structures were designed for miRNA cDNA synthesis based on a previously published article (22-24). The sequences of primers are presented in Tables 1 and 2.

***RNA extraction and cDNA synthesis***

Total RNA was extracted using RNX^®^-Plus (Cinnagen, Tehran, Iran) according to the manufacturer’s instruction. The quantity of the RNA was determined and then it was divided in half for mRNA and miRNA expression analysis. Using the pooled sample of 15 healthy samples, the quality and quantity of the extracted RNA were evaluated. Considering the problems of obtaining normal lymphoblast-containing bone marrow samples, peripheral blood mononuclear cells (MNCs), which are mostly T lymphocytes, were used as controls (25).

cDNA synthesis for target genes was performed using RevertAid^®^ First Strand cDNA Synthesis Kit (Themoscientific, USA, Cat. No. K1622) and random hexamers. For miRNA cDNA synthesis, stem-loop primers were used as previously described (24).

***Quantitative Real-Time PCR (RT-qPCR)***

Relative RT-qPCR was performed in StepOne™ instrument (ABI, USA). The reactions included 10 µl RealQ Plus 2X master mix Green (Amplicon, Denmark), 0.4 µM each primer, 0.4 µl 50X ROX dye, 2 µl cDNA, and 6.8 µl distilled water in a final volume of 20 µl. After 40 cycles, a melt curve analysis was performed. After performing Real-Time PCR, threshold cycles were determined and 2^-∆∆CT^ method was used to analyze the final results. All tests were performed in duplicates. *β-actin* and *SNORD47* were used as housekeeping genes for mRNAs and miRNAs expressions, respectively.

***Statistical analysis***

The data were analyzed using SPSS statistical software package (version 22). All the results were expressed as mean±Standard error. For all of the analyses, a two-sided *P*-value of less than 0.05 was considered statistically significant. The efficiency of Real-Time PCR reactions were determined in LinReg and entered in REST^®^ 2009 software for final analyses.

## Results

***Bioinformatics predictions***

Using TargetScan, PicTar, Miranda, DIANA microT CDS, miRBase, and miRWalk databases, miRNAs targeting *NOTCH1*, *c-Myc*, and *CCND1* mRNAs were determined. The results were sorted in MS Excel application. Then, among 2000 predicted miRNAs, those that were predicted in at least three of the databases and targeting all three genes were selected, which included miR-34a and miR-449a. miR-1827 and miR-106b were also considered for further studies since they had high miRNA: mRNA attachment energy (Table 3).

***Target mRNAs and targeting miRNAs gene expression in Jurkat cell line***

**Figure 1.**
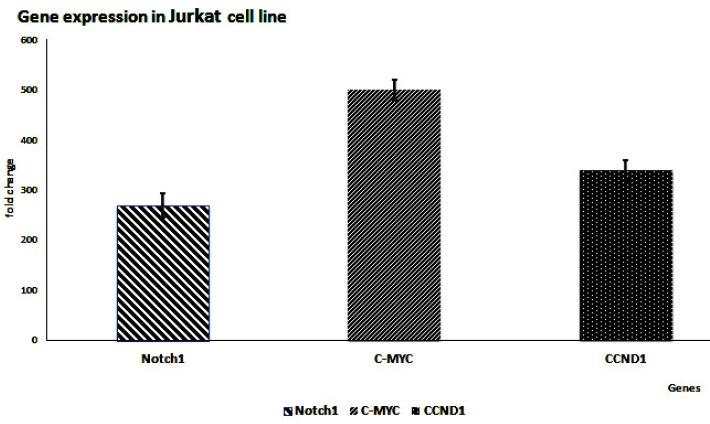
Expression level of Notch1, c-Myc, and CCND1 mRNAs in Jurkat cells

**Figure 2.**
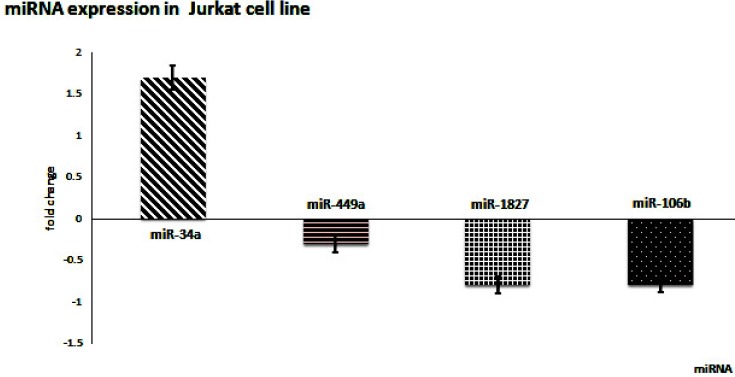
Expression level of miR-449a, miR-106b, miR-1827, and miR-34a in Jurkat cells

RT-qPCR results showed that all three genes were up-regulated in the cell line. In addition, miR-449a, miR-106b, and miR-1827 were down-regulated, while miR-34a was slightly up-regulated. These results confirmed our hypothesis of inverse correlation between the miRNAs and the mRNAs’ expression save miR-34a (Figures 1 and 2).

***Target mRNAs and targeting miRNAs gene expression in clinical samples***

**Figure 3:**
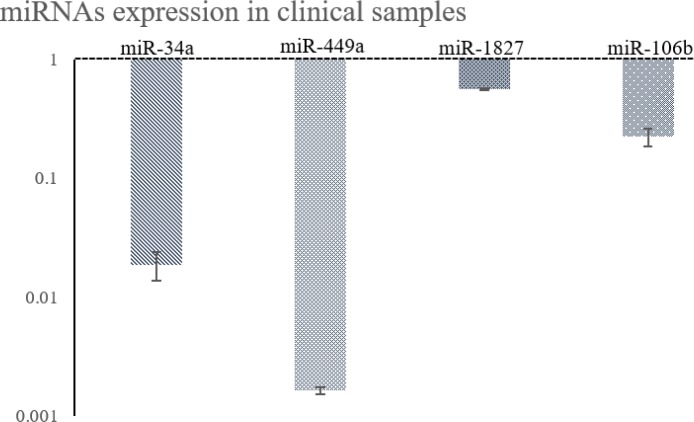
Expression pattern of the miRNAs in T-cell acute lymphoblastic leukemia (T-ALL) clinical samples

**Figure 4.**
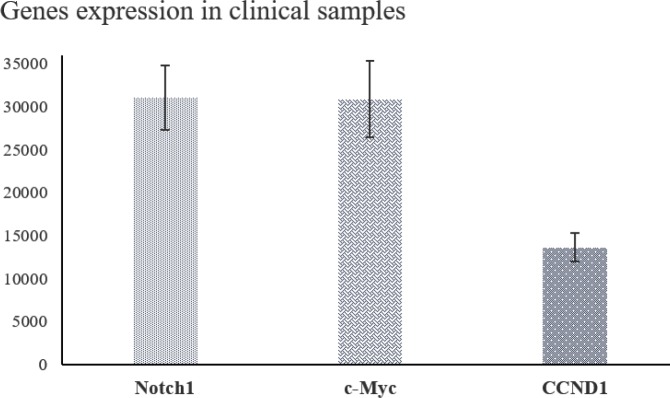
Expression pattern of the genes in T-cell acute lymphoblastic leukemia (T-ALL) clinical samples

The expression of *NOTCH1* and *c-Myc* showed increase in all twenty clinical samples compared to the normal samples. Furthermore, *CCND1* was up-regulated in 80% of samples. On the other hand, miR-34a and -449a, miR-106b, and miR-1827 were down-regulated in 100%, 95%, and 80% of cases, respectively. These results indicate that most samples with increased expression of the genes had decreased expression of the miRNAs (Figures 3 and 4). 

Using Spearman Correlation test, the correlation coefficient for the miRNAs and their target genes was shown to be negative, i.e. they are inversely correlated. Correlation coefficient for miR-34a-Notch1, miR-34a-c-Myc, and miR-34a-CCND1 was determined to be -1, -1, and -0.8, respectively (*P*-value< 0.05). In addition, correlation coefficient for miR-449a-Notch1 and miR-449a-CCND1 was determined to be -1 and -0.8, respectively (*P*-value< 0.05). We also detected this correlation factor to be -0.8 and -0.75 for miR-1827-c-myc and miR-106b-CCND1, respectively (*P*-value= 0.05). 

## Discussion

T-ALL is a malignancy of immature T lymphocytes and its prognosis is worse than that of B-ALL. In addition, another concern is its drug resistance and poor drug response. In fact, the only treatment strategy for patients diagnosed with T-ALL is chemotherapy, and there is no need to mention its complications and side effects. Therefore, using advanced diagnostic and monitoring approaches are the most important challenges ahead (26-28). One of such advanced methods that has recently drawn scientists’ attention is miRNAs, which are small non-coding molecules that can regulate gene expression and play an important role in various diseases such as cancers. The first evidence emphasizing on their role in cancer was documented in 2002 and showed that miRNA genes located at fragile and deleted segments of cancer genes. It was postulated that these deletions have a principal role in cancer pathogenesis (29). Later, other studies indicated that miRNAs have a pivotal role in diseases other than cancer like viral infections, cardiovascular diseases, and inflammation (30). Hence, using these biomolecules as biomarkers and therapeutic targets can be a potent tool in disease treatment.

The role of miRNAs in T-ALL etiology and pathogenesis has been gradually proved. In 2011, Mavrakis and colleagues studied 50 clinical samples and 18 T-ALL cell lines and found out that miR-20a, miR-92, miR-26a, miR-223, and miR-19b had been increased in T-ALL and subsequently suppressed several tumor suppressor genes including *IKAROS*, *PTEN*, *BIM*, *PHF6*, *NF1*, and *FBXW7*. These miRNAs were able to induce leukemia in mice within 75 days (31). On the other hand, Lv *et* *al*. in 2012 showed that miR-142-3p was a biomarker and therapeutic target for T-ALL (32). 

Notch signaling is a vital pathway in T-ALL, and mutations in the pathway’s genes have been determined in 60% of the cases. To evaluate the relationship between this pathway and miRNAs, Gusscott *et* *al*. inhibited the pathway using Gamma secretase and found that the expression of miR-223 increased. In another word, activation of Notch pathway suppresses miR-223 (33). Li and colleagues also studied the relationship between miR-451, miR-709, and *MYC* as a down-stream gene in Notch pathway. They concluded that these miRNAs directly suppressed *MYC* and were oncogene suppressors in a mouse model of T-ALL. Their results indicated that repression of these tumor suppressor miRNAs is necessary to induce T-ALL (34).

In the present study, we tried to assess the expression of miR-34a, miR-1827, miR-449a, and miR-106b, which were selected by bioinformatics approaches, and three principal genes of Notch signaling pathway, i.e. *NOTCH1*, *c-Myc*, and *CCND1*, in a T-ALL cell line and clinical samples.

Bioinformatics approaches are less costly and more available than other methods. In addition, they can effectively reveal various aspects of miRNA biology. These tools contain a wide spectrum of data related to miRNA genes and their predicted targets that have key roles in their function. These databases and algorithms can predict miRNA targets based on the sequence of target 3’-UTRs or the possibility of binding to seed regions (35). Nonetheless, they have some limitations including false positive results. However, it can be overcome by simultaneous use of several algorithms.

Based on our results, *NOTCH1*, *c-Myc*, and *CCND1* were overexpressed in most of the clinical samples as well as Jurkat cell line. 

Since it was previously confirmed that *NOTCH1* was up-regulated in T-ALL samples, and *c-Myc* is an important down-stream target of Notch signaling, these results were not surprising.

*NOTCH1*, as the main gene and promoting factor of the pathway, affects several down-stream genes such as *c-Myc*, which has a role in cell growth and proliferation, cell survival, angiogenesis, stem cell self-renewal, cell metabolism, and drug resistance. In addition, it is implicated in induction of T-ALL development, lymphomas, ovarian cancer, and so on (36-38). *CCND1*, encoding Cyclin-D1, plays a part in cell cycle and induction of G1 phase and progression of S-phase. Any dysregulation in expression of this gene results in an increased risk for cancer development (17). In the present study, 100% increase observed in *NOTCH1* and *c-Myc *expressions in clinical samples shows their pivotal role in Notch signaling and the effect of their dysregulation in T-ALL progression. Since *CCND1* increased in 80% of the cases, it seems that its expression is also affected by other miRNAs and regulatory genes. 

Expression analysis of the selected miRNAs showed that they were up-regulated in the clinical samples. miR-34a and -449a were down-regulated in 100% of cases, while miR-106b and miR-1827 showed decline in 95% and 80% of samples, respectively. Results less than 100% seem to originate from bioinformatics prediction errors, which can be minimized using several algorithms simultaneously.

In Jurkat cells, miRNAs were down-regulated except for miR-34a. It can be concluded that the behavior, gene and miRNA expression of cell lines can change with repeated passages *in vitro*.

 Considering the expression of the genes and the miRNAs in clinical samples, we observed an obvious inverse correlation between *NOTCH1*/miR-34a, *c-Myc*/miR34a, *CCND1*/miR-34a (in 100%, 100%, and 80% of cases, respectively), miR-449a/* NOTCH1*, miR-499a/*CCND1* (in 100% and 80% of the cases, respectively), miR-1827/*c-Myc*, and miR-106b/*CCND1* (in 80% and 75% of the cases, respectively) (*P* -values≤ 0.05). This phenomenon demonstrates tumor suppressive role of the selected miRNAs in T-ALL.

Tumor suppressive role of miR-34a was first discovered during a study on *c-Myc*, *LDHA*, and *SIRT1* oncogenes. In addition, in several researches, its tumor suppressive role on *NOTCH1* (apoptosis induction and proliferation inhibition) and *c-Myc/CCND1* (G1 arrest) has been reported (39-41). In other studies, *NOTCH1* was reported to be the direct target of miR-449a. It is also confirmed that miR-449a is a tumor suppressor in neuroblastoma, myeloid leukemia, celiac disease, and gastric cancer (42-44). MiR-1827 is also reported to be a tumor suppressor by regulating P53 (45) and miR-106b, and is a potent biomarker for gastric cancer, hepatocellular carcinoma, chronic hepatic disease, and cutaneous melanoma (46, 47). 

All these studies along with the present research confirm the tumor suppressive role of most of the selected miRNAs; however, more investigation in a larger sample size is required. Finally, considering all limitation of this study, we propose that these miRNAs can be considered as targets and biomarkers for future diagnostic and therapeutic approaches.

All these studies along with the present research confirm the tumor suppressive role of most of the selected miRNAs; however, more investigation in a larger sample size is required. Finally, considering all limitation of this study, we propose that these miRNAs can be considered as targets and biomarkers for future diagnostic and therapeutic approaches.

**Table 1:** The sequence of primers used for the analysis of the genes expression

**Gene Name**	**Forward Primer**	**Reverse Primer**
Beta Actin	CTTCCTTCCTGGGCATG	GTCTTTGCGGATGTCCAC
NOTCH1	CTGGTCAGGGAAATCGTG	TGGGCAGTGGCAGATGTAG
c-MYC	GCGACTCTGAGGAGGAAC	CTGCGTAGTTGTGCTGATG
CCND1	ATCTACACCGACAACTCCATC	TGTTCTCCTCCGCCTCTG

**Table 2:** The sequence of Reverse-Transcription (RT)-stem-loop primers and sense primers for miRNA Real-time PCR

**Sequence**	**miRNA**
5’ UGGCAGUGUCUUAGCUGGUUGU 3’	hsa-miR-34a
5’ GGT CGT ATG CAG AGC AGG GTC CGA GGT ATC CAT CGC ACG CAT CGC ACT GCA TAC GAC CACAACC 3’	miR-34a RT Stem-loop primer
5’ AGGGTG GCA GTGTCTTAGC 3’	miR-34a Forward primer
5’ UGGCAGUGUAUUGUUAGCUGGU 3’	hsa-miR-449a
5’GGTCGTATGCAGAGCAGGGTCCGAGGTATCCATCGCACGCATCGCTCTGCATACGACCACCAG 3’	miR-449a RT Stem-loop primer
5’ GGCGTGGCAGTGTATTGTTAG 3’	miR-449a Forward primer
5’ UGAGGCAGUAGAUUGAAU 3’	has-miR-1827
5’GGTCGTATGCAGAGCAGGGTCCGAGGTATCCATCGCACGCATCGCTCTGCATACGACCATTCA 3’	miR-1827 Stem-loop primer
5’ GGGCGTGAGGCAGTAGAT 3’	miR-1827 Forward primer
5’ UAAAGUGCUGACAGUGCAGAU 3’	has-miR-106b
5’GGTCGTATGCAGAGCAGGGTCCGAGGTATCCATCGCACGCATCGCTCTGCATACGACCATCTG 3’	miR-106b Stem-loop primer
5’ GGGCTAAAGTGCTGACAGTG 3’	miR-106b Forward primer
5’ GAG CAG GGT CCG AGG T 3’	Universal Reverse

**Table 3:** Scoring table obtained from miRNA bioinformatics databases

**SUM**	**PicTar**	**Targetscan**	**MirWalk validate**	**MirWalk**	**MiR** **anda**	**DIANA** **CDS**	**DIANA microT**	**Gene name**	**microRNA**
5	+	+	+		+	+		Notch-1	miR-34a
4	+	+	+		+			c-Myc	
3	+		+		+			CCND1	
4		+	+		+	+		Notch-1	miR-449 a
1					+			c-Myc	
2			+		+			CCND1	
4		+		+		+	+	c-Myc	miR-1827
6	+	+	+		+	+	+	CCND1	miR-106b

## Conclusion

In the present study, considering the role of Notch signaling pathway and miRNAs in T-ALL, we aimed to evaluate the most specific genes of Notch pathway and their specific targeting miRNAs. By analyzing data obtained from bioinformatics methods and databases, an inverse correlation between NOTCH1, c-Myc, and CCND1 and their targeting miRNAs was considered to be tested in T-ALL clinical samples. By statistically comparing the expression results and finding reverse correlation between the expression of the genes and target miRNAs, it seems they can be used as effective biomarkers for diagnosis and as therapeutic targets after implementing necessary measures.
